# A screen of repurposed drugs identifies AMHR2/MISR2 agonists as potential contraceptives

**DOI:** 10.1073/pnas.2122512119

**Published:** 2022-04-05

**Authors:** Yi Li, Lina Wei, Marie-Charlotte Meinsohn, Rana Suliman, Maeva Chauvin, Jim Berstler, Kate Hartland, Mark M. Jensen, Natalie A. Sicher, Nicholas Nagykery, Patricia K. Donahoe, David Pepin

**Affiliations:** ^a^Pediatric Surgical Research Laboratories, Massachusetts General Hospital, Boston, MA 02114;; ^b^Department of Surgery, Harvard Medical School, Boston, MA 02115;; ^c^Center for the Development of Therapeutics (CDoT), Broad Institute of Massachusetts Institute of Technology and Harvard, Cambridge, MA 02142

**Keywords:** contraception, AMHR2 agonists, small molecules screen, AMH, folliculogenesis

## Abstract

We identified the anti-Mullerian hormone (also known as Müllerian inhibiting substance or MIS) as an inhibitory hormone that induces long-term contraception in mammals. The type II receptor to this hormone, AMHR2 (also known as MISR2), represents a promising druggable target for the modulation of female reproduction with a mechanism of action distinct from steroidal contraceptives. We designed an in vitro platform to screen and validate small molecules that can activate MISR2 signaling and suppress ovarian folliculogenesis. Using a bone morphogenesis protein (BMP)–response element luciferase reporter cell–based assay, we screened 5,440 compounds from a repurposed drug library. Positive hits in this screen were tested for specificity and potency in luciferase dose–response assays, and biological activity was tested in ex vivo Mullerian duct regression bioassays. Selected candidates were further evaluated in ex vivo follicle/ovary culture assays and in vivo in mice and rats. Here, we report that SP600125, CYC-116, gandotinib, and ruxolitinib can specifically inhibit primordial follicle activation and repress folliculogenesis by stimulating the MISR2 pathway.

## Significance

This study aims to identify drugs that activate the Mullerian inhibiting substance pathway to be used for contraception or other applications in women’s health. We describe a high-throughput screening pipeline to identify small molecules that activate the Mullerian inhibiting substance type 2 receptor (MISR2) and validate their activity in bioassays. We identify five compounds from a repurposed drug library that specifically induce MISR2 signaling, trigger regression of the Mullerian duct, and inhibit follicle activation. We test these compounds in vivo and show that they can repress folliculogenesis in mice and rats in an *Misr2*-dependent manner. These drugs may represent a class of ovarian regulators that inhibit preantral follicle activation and growth.

**Fig. 1.**
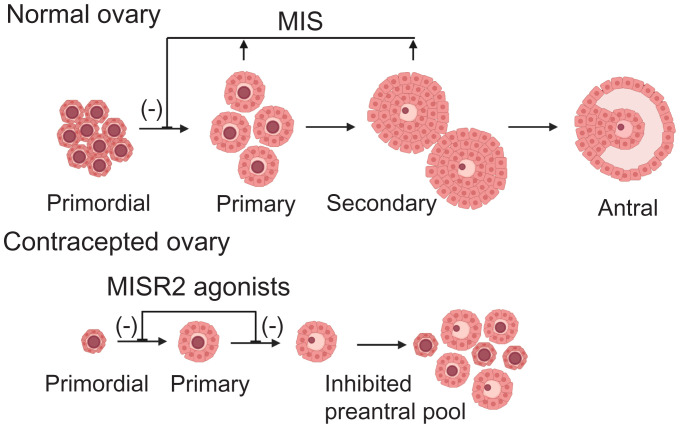
Model of the mechanism of action of MIS and MISR2 agonists in folliculogenesis.

Women are born with a finite number of primordial follicles, and their activation triggers an irreversible process that ends in either ovulation or follicular atresia and continues until the primordial follicle pool is depleted at menopause (1, 2). The anti-Mullerian hormone (AMH), also known as Mullerian inhibiting substance (MIS), is a paracrine glycoprotein ligand of the Transforming growth factor beta (TGFb) superfamily produced by granulosa cells of growing follicles, which regulate ovarian reserve maintenance by providing negative feedback to primordial follicle activation (Fig. 1) (3). AMH/MIS is unique in the TGFb superfamily for having a one-to-one specificity with its type II receptor, AMHR2, also known as MISR2 (4). We have previously shown that administration of exogenous MIS at superphysiological levels in mice profoundly inhibits folliculogenesis and results in complete contraception (5, 6) as MIS inhibits both the activation and differentiation of preantral follicles (7). This suppression of follicles is reversible upon cessation of MIS treatment (8). In a proof-of-concept study using gene therapy with an adeno-associated viral vector to deliver a recombinant MIS, female mice secreted superphysiological levels of MIS and became sterile and acyclic but maintained detectable sex steroid production (5). We have therefore identified the initial activation phase of primordial follicles and their early maturation as preantral follicles as a prime target for pharmacological disruption for the purpose of ovarian suppression. This mechanism of contraception is unlike all current forms of hormonal contraception that regulate folliculogenesis at the antral stage and prevent ovulation by disrupting the hypothalamic-pituitary-gonadal axis (9).

Given the clinical potential of a highly potent, nonsurgical, nonhormonal, reversible inhibitor of ovarian follicle development that prevents cycling but maintains healthy estrogen levels, we sought to screen small-molecule drugs acting upon the MISR2 pathway that could potentially be translated to the clinic. Previous work had successfully employed an in vitro assay based on a concatemerized BMP response element derived from the Id1 promoter driving the expression of luciferase (BRE-luc) to identify both BMP receptor 2 and MISR2 agonists (10, 11). In the latter study, the BRE-luc was cotransfected with either the wild-type MIS receptor (*MISR2*) or a catalytically inactive version (*MISR2* K228R) in COS7 cells, which otherwise lack endogenous MISR2, and identified specific agonists of MISR2. Using this strategy to screen a small compound library, we previously identified the anthropyrazolone SP600125 as an MISR2 agonist (10).

Herein, we report that following improvements to the reporter assay and the screening of a larger drug repurposing library with 5,440 compounds, we have identified nine candidate MISR2 agonists. We validated the activity of those nine candidates in secondary assays and chose four (SP600125, CYC-116, gandotinib, and ruxolitinib) for further evaluation in tertiary bioassays and in vivo in mice, culminating with the confirmation of their ability to produce ovarian suppression.

## Results

### Optimization of a BRE-Luc Reporter Assay and Its Use in a Small-Molecule Screen.

**Fig. 2.**
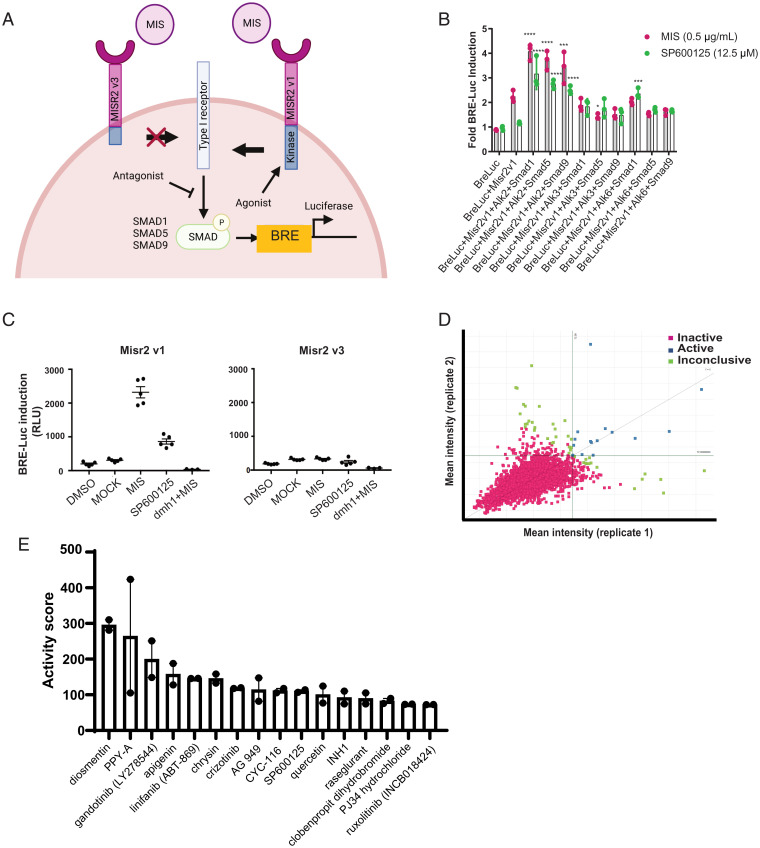
(*A*) Schematic of MISR2 signaling pathway and luciferase reporter. (*B*) Luciferase reporter optimization by transfection of combinations of type I receptors (*ALK2/3/6*) and receptor SMADs (*SMAD1/5/9*) with BRE-luc and *MISR2v1* constructs. Luminosity was measured 24 h following treatment with MIS (0.5 µg/mL) or SP600125 (12.5 µM) and plotted relative to vehicle control (*n* = 3 per treatment, mean ± SD, *P < 0.05, ***P < 0.005, ****P < 0.001). (*C*) Comparison of luciferase induction in COS7 transiently transfected with BRE-luc, ALK2, SMAD1, and MISR2v1 or MISR2v3 and treated with MIS (10 µg/mL), SP600125 (12.5 µM), DMH1 (10 µM) + MIS (10 µg/mL), or equivalent volume of mock-purified MIS (MOCK) as a control for MIS or vehicle control (DMSO) for SP600125 (*n* = 5 per group, mean ± SD). (*D*) Scatterplot of luciferase intensity values across two replicates. *Right* quadrant identifies hits with 3 SD induction across both replicates. (*E*) Active hits identified across two replicates ranked by median activity score (*n* = 2 replicates, mean ± SD).

To identify agonists of MISR2, we used a transcriptional reporter based on a BMP response element driving luciferase transcription (BRE-luc) in COS7 cells, which are normally devoid of MISR2, as previously described (10). We hypothesized that in addition to the introduction of the active human MISR2 splice variant 1 (*MISR2v1*), the added overexpression of other downstream effectors of the MISR2 signaling pathway could increase the sensitivity of that assay (Fig. 2*A*). Thus, we evaluated cotransfections with all three human type I receptors known to interact with *MISR2*, namely activin receptor-like kinases *ALK2*(*ACVR1*), *ALK3* (*BMPR1a*), and *ALK6* (*BMPR1b*), along with the three human receptor SMADs known to be phosphorylated by these receptors, namely *SMAD1*, *SMAD5*, and *SMAD9* (Fig. 2*B*) (12).

A combinatorial approach was employed, in which we transfected COS7 cells with *ALK2* + *SMAD1/5/9*, *ALK3* + *SMAD1/5/9*, or *ALK6* + *SMAD1/5/9*, in addition to *MISR2* and BRE-luc, using equal amounts of each plasmid to identify the optimal combination. While transient cotransfection of *MISR2* with BRE-luc alone resulted in a 2.25 ± 0.24-fold induction of the luciferase signal following treatment with MIS compared to vehicle control, the addition of *ALK2* significantly increased the signal, peaking with the *ALK2*/*SMAD1* combination at 4.07 ± 0.33-fold over vehicle control using MIS (0.5 µg/mL) and 3.15 ± 0.64-fold using SP600125 (12.5 µM) (Fig. 2*B*). To confirm that the induction of luciferase was dependent on MISR2 activation, we designed a counter-screen consisting of a COS7 cell line transfected with all the same pathway components (*ALK2*, *SMAD1*, BRE-luc), but replacing *MISR2v1* with a naturally-occurring inactive splice variant, *MISR2v3*, which has no kinase activity (13) (Fig. 2*A*). Following cotransfection of COS7 cells with *MISR2v3*, *ALK2*, *SMAD1*, and BRE-luc, no significant luciferase induction was seen upon treatment with MIS or SP600125 (Fig. 2*C*). Moreover, we demonstrated by qPCR and immunofluorescence that the *MISR2v3* inactive splice variant is transcribed, translated, and localized similarly to the active full-length *MISRv1* (*SI Appendix*, Fig. S1 *A* and *B*). To further confirm that in this assay the agonism of MISR2 results in the activation of *ALK2*, we treated the transfected cell with the potent ALK2 inhibitor, the dorsomorphin homolog 1(DMH1) (14), which resulted in the abrogation of luciferase induction upon MIS treatment (Fig. 2*C*).

We used this optimized protocol (BRE-luc, *MISR2*, *ALK2*, *SMAD1*) to transiently transfect COS7 cells, which were then plated in 384 well plates. The wells in these plates were individually treated with 5,440 compounds dissolved in dimethylsulfoxide (DMSO) from a drug-repurposing library of the Broad Institute, containing approved drugs, drugs in clinical trials, and preclinical compounds (15). Following a 24 h incubation with these drugs at 50 µM, a luciferase substrate was added and luminescence was measured. This screen was performed twice. We identified 16 candidates (Fig. 2*D* and *E*) with robust and reproductible activation of the reporter with a cutoff defined as 3 SDs from the mean vehicle control (DMSO) in both replicates. Candidate compounds were ranked by activity score, revealing a previously known MISR2 agonist, SP600125, as the 10th most active compound (Fig. 2*E*) (10). To evaluate the relatedness of the active compounds we used the Tanimoto structural correlation, which suggested a close relationship of the flavonoid compounds such as apigenin, luteolin, diosmetin, and chrysin (*SI Appendix*, Fig. S2*A*).

Principal component analysis based on these scores also suggested structural similarities between the pairs linifanib/PPY-A and CYC-116/gandotinib/ruxolitinib (*SI Appendix*, Fig. S2*B*).

### Secondary Validation of Hits and Related Chemical Entities.

To validate the activity of positive hits and ensure the specificity of the response, we carried out a secondary validation screen in which we evaluated a range of concentrations of each compound in the reporter COS7 cells transfected with either the active *MISRv1* or inactive *MISR2v3* splice variants. The luminescence dose–response of drugs spanned a 10-point range of concentrations from 0.2 µM to 100 µM.

**Fig. 3.**
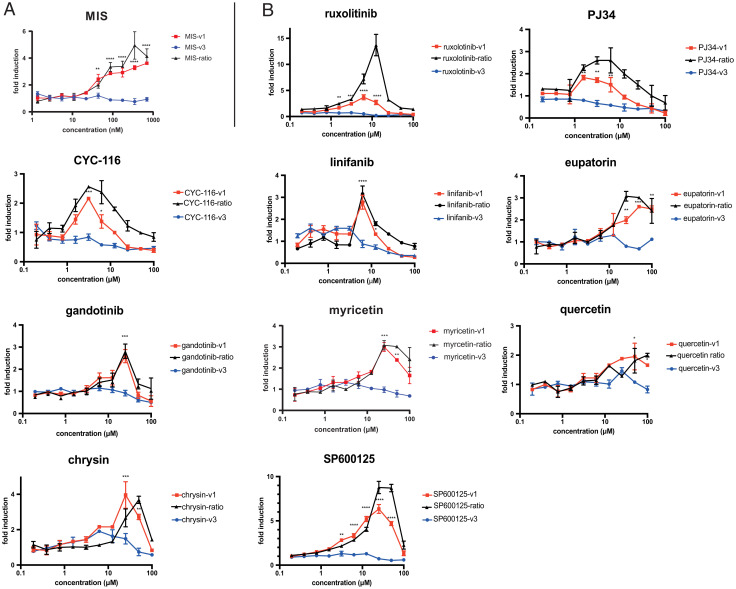
Dose–response validation of MISR2 agonist candidates and related chemical entities. Luciferase induction compared to vehicle control is plotted across a 10-point range of (*A*) MIS (1.36–700 nM) or (*B*) compounds (195 nM–100 µM), in COS7 cells transfected with either MISR2v1 in red or MISR2v3 in blue (in addition to ALK2/SMAD1/BRE-luc) and incubated for 24 h. The MISR2v1/MISR2v3 signal ratio is also plotted in black (*n* = 2 replicates, mean ± SEM, *P < 0.05, ***P < 0.005).

By measuring the ratio of fold induction of luminescence in *MISR2v1-* versus *MISR2v3*-transfected cells, we could normalize for toxicity and ensured that the response was dependent on MISR2 activity. We tested all 16 identified candidates from the primary screen, along with 7 closely related compounds that were not part of the 5,440 compounds from the repurposed drug library. The extra compounds (eupatorin, myricetin, sinensetin, nobiletin, quercitrin, kaemferol) were chosen because of their close structural homology to the flavonoids apigenin, luteolin, diosmetin, and chrysin identified as hits in the primary screen. This approach was validated using the fold ratio of *MISR2v1*/*MISR2v3* with the recombinant Albumin Leader Q425R Mullerian Inhibiting Substance (LRMIS) protein (16), which only showed significant dose-dependent induction of luciferase activity with *MISR2v1* (Fig. 3*A*). We confirmed a statistically significant induction of luminescence in *MISR2v1*-transfected cells, but not *MISR2v3*-transfected cells, in 10 of the 16 compounds: linifanib, CYC-116, chrysin, SP600125, ruxolitinib, gandotinib, PJ34, quercetin, eupatorin, and myricetin (Fig. 3*B*) but not diosmetin, PPY-A, apigenin, cryzotinib, AG-494, ADX-10059, clobenpropit, luteolin, or Inh1 (*SI Appendix*, Fig. S3*A*). Only compounds with significant luminescence induction at 25 µM or less were selected for further evaluation in tertiary validation assays. The induction of luciferase in a dose–response counter screen of hits that were not validated as positive (*SI Appendix*, Fig. S3*A*) and additional compounds chemically related to the hits that were not validated as positive (*SI Appendix*, Fig. S3*A*), as well as the chemical structure of the validated hits (*SI Appendix*, Fig. S3*C*), can be seen in *SI Appendix*, Fig. S3.

### Tertiary Validation of Hits in Biological Assays.

**Fig. 4.**
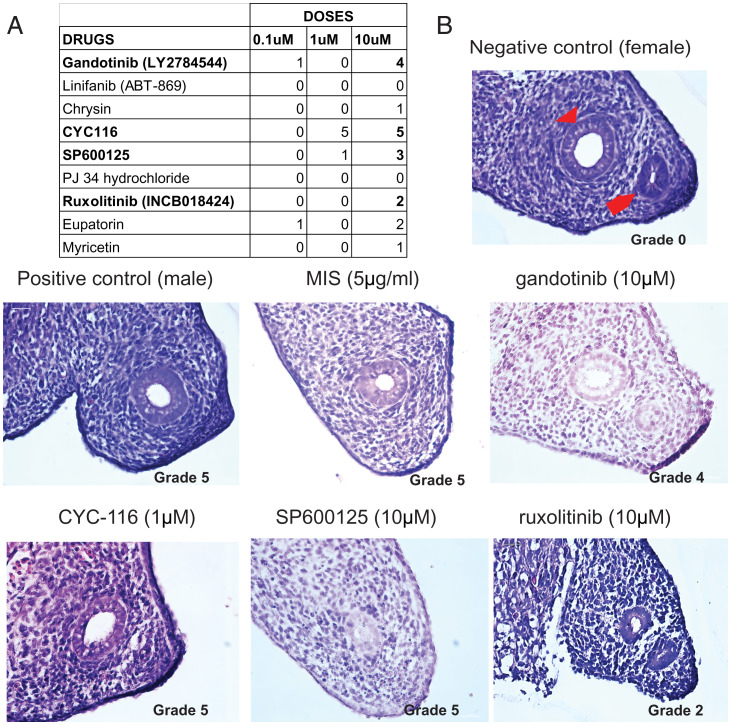
Ex vivo tertiary bioassays to validate specific activities of candidate MISR2 agonists. (*A*) Table of median Mullerian regression grade, where a score of 0 indicates no regression (negative control, female ridge) and 5 indicates full regression (positive control, male urogenital ridge) for compounds tested at least 3 times at 0.1, 1, and 10 μM (*n* = 3–5 per treatment). (*B*) A representative H&E-stained section of a female fetal rat urogenital ridge considered as a negative control (female ridge [grade 0], Wolffian duct designated by red arrow head and Mullerian duct by red arrow) or a positive control male ridge (male ridge [grade 5]), or female ridges treated ex vivo with 0.1 to 10 μM of MIS, gandotinib, ruxolitinib, SP600125, and CYC-116 diluted in media for 72 h.

To ensure both the specificity and the potency of the biological effects of candidate MISR2 agonists, we used a gold-standard bioassay of MIS activity, the rat fetal urogenital ridge Mullerian duct regression assay (17). Compounds were empirically tested at 0.1 µM, 1 µM, and 10 µM to assess a probable activity/toxicity window. We tested SP600125, gandotinib, chrysin, linifanib, CYC-116, eupatorin, PJ34, myricetin, and ruxolitinib at least 3 times at each dose. Regression of the Mullerian duct was graded from 0 (no regression) to 5 (complete regression) and only compounds with a median score superior or equal to 3 in the three replicates with minor toxicity were considered as active. We were able to detect major to complete regression with minimal toxicity with gandotinib (10 µM), SP600125 (10 µM), and CYC-116 (1 µM) (Fig. 4). Although ruxolitinib showed only partial regression at 10 µM, with grade 2, it was selected for further analysis based on its high structural homology with gandotinib (Fig. 4 and *SI Appendix*, Fig. S3*C*). We therefore chose these compounds for further evaluation in bioassays relevant to their target activity in the ovaries.

To further ensure that candidate MISR2 agonists could elicit the same canonical downstream signaling as MIS in ovaries, we treated cultured mouse ovaries with gandotinib (1 µM), SP600125 (1 µM), CYC-116 (1 µM), ruxolitinib (1 µM), MIS as a positive control (10 µg/mL), or DMSO as a vehicle control (equal volume) for 48 h. These lower doses were selected due to the relatively poor tolerance of ovaries cultured ex vivo to cytotoxic compounds (18).

**Fig. 5.**
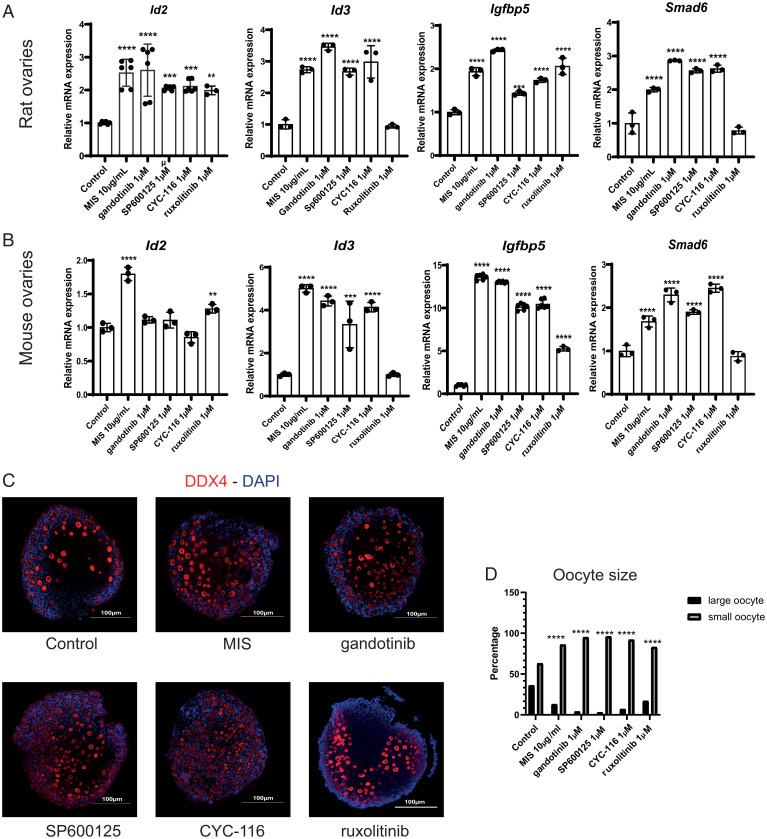
Evaluation of putative MISR2 agonists for their ability to recapitulate an MIS response in cultured ovaries. (*A*, *B*) The induction of MIS target genes *Id2*, *Id3*, *Smad6*, and *Igfbp5* was recapitulated as measured by qPCR following a 48 h incubation of ex vivo cultured ovaries from PND2 rats (*A*) or mice (*B*) with 1 μM candidate MISR2 agonists gandotinib, SP600125m, CYC-116, ruxolitinib, MIS at 10 μg/mL as a positive control, and DMSO as a vehicle control (*n* = 3 per treatment, mean ± SD, **P < 0.01, ***P < 0.005, ****P < 0.001). (*C*, *D*) Representative DDX4 immunofluorescence (*C*) and oocyte size distribution in percentage (*D*) following 48 h incubation of ovaries from PND2 mice cultured ex vivo with 1 μM candidate MISR2 agonists gandotinib, SP600125, CYC-116, ruxolitinib, MIS at 10 μg/mL as a positive control, and DMSO as a vehicle control (oocyte counted from 1 representative middle section, activated/large oocyte threshold 12 μm and above, *n* = 4 per treatment, ****P < 0.001).

We then evaluated the ability of these compounds to recapitulate the same changes in gene expression previously identified in granulosa cells treated with MIS, such as the induction of *Id2*, *Id3*, *Smad6*, and *Igfbp5* as measured by qPCR (7). We found that most target genes were significantly induced in cultured rat and mouse ovaries by all four candidate agonists, except for ruxolitinib with *Id3* and *Smad6* in mice and rats (Fig. 5*A* and *B*). Furthermore, we confirmed by RNA in situ hybridization (RNAish), using RNAscope probes, that *Id3* and *Igfbp5* were being induced in granulosa cells of primordial follicles in the cultured mouse ovaries (*SI Appendix*, Fig. S4*B*). In contrast, we found that *Misr2* itself was upregulated only by MIS treatment but not any of the MISR2 agonists by both qPCR (*SI Appendix*, Fig. S4*A*) and RNAish (*SI Appendix*, Fig. S4*B*). Moreover, in the mouse ovaries, we uncovered a significant decrease of *Nr5a2*, an orphan nuclear receptor specifically expressed in the granulosa cells of all active follicles from the primordial stage forward upon treatment with MIS and all four candidate agonists as seen by qPCR (*SI Appendix*, Fig. S4*A*) (19). In contrast, the expression of the germ cell marker *Nobox* was unchanged by treatment with any of the candidates or MIS, as analyzed by qPCR, suggesting that the abundance of germ cells was unaffected by those drugs (*SI Appendix*, Fig. S4*A*).

To evaluate the ability of the candidate MISR2 agonists to inhibit preantral follicle growth, we treated ex vivo postnatal day 2 (PND2) mouse ovarian cultures with gandotinib (1 µM), SP600125 (1 µM), CYC-116 (1 µM), ruxolitinib (1 µM), MIS as a positive control (10 µg/mL), or vehicle control (DMSO) for 48 h. Ovaries were then sectioned and oocytes were detected by immunofluorescence with an anti-DDX4 antibody (Fig. 5*C*), and their diameters were measured in micrographs of the four largest (middle) sections using Image J software. Using an oocyte diameter threshold of <12 µM, we estimated the number of quiescent primordial follicles (20), while all those above that threshold were considered growing preantral follicles. We found that all treatments examined resulted in a significantly lower proportion of large oocytes to small oocytes than DMSO controls, suggesting the inhibition of primordial follicle activation and growth (Fig. 5*D*). To ensure that the inhibitory effect of candidate MISR2 agonists on activation was generalizable to all primordial follicles and not just in the first wave of follicle formation and activation after birth, we mechanically dissociated adult mouse ovaries and manually retrieved intact individual primordial follicles. Those follicles were cultured in droplets, a method routinely used for preimplantation embryo cultures, and were treated with gandotinib (1 µM), SP600125 (1 µM), CYC-116 (1 µM), ruxolitinib (1 µM), MIS as a positive control (10 µg/mL), and DMSO as a vehicle control for 48 h. Following culture, viability was verified using Calcein dye, and follicles (*n* >33 per condition) were fixed and analyzed by immunofluorescence with an anti-INHA antibody (*SI Appendix*, Fig. S5*A*), a marker of differentiating granulosa cells, and assessed morphologically based on the appearance of the granulosa cell layer (squamous versus cuboidal) (*SI Appendix*, Fig. S5*A*, brightfield) to categorize them as activated (INHA^high^, cuboidal) or quiescent (INHA^low^, squamous) (*SI Appendix*, Fig. S5*B*). All treatments examined resulted in a significantly lower proportion of activated follicles, with a greater than 50% reduction compared to DMSO controls, suggesting again the inhibition of primordial follicle activation (*SI Appendix*, Fig. S5*B*).

### In Vivo Treatments with MISR2 Agonists Result in Significant Suppression of Ovarian Folliculogenesis.

**Fig. 6.**
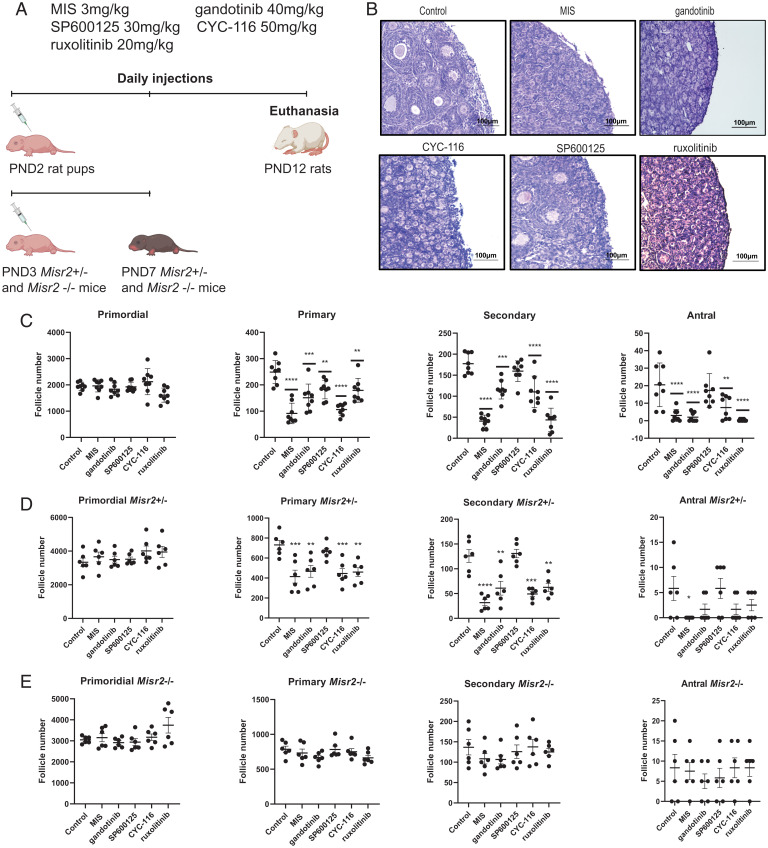
In vivo ovarian suppression following treatment with MISR2 agonists. (*A*) Schematic representation of in vivo experiments in rats and Misr2 knockout mice injected daily from PND2 to PND12 and from PND3 to PND7, respectively, with gandotinib (40 mg/kg), SP600125 (45 mg/kg), CYC-116 (50 mg/kg), ruxolitinib (20 mg/kg), or vehicle control. (*B*) Representative H&E-stained sections of ovaries from PND2 rat pups treated once a day for 10 d with gandotinib (40 mg/kg), SP600125 (45 mg/kg), CYC-116 (50 mg/kg), ruxolitinib (20 mg/kg), or vehicle control (*n* = 8 per group), and (*C*) corresponding follicle counts (mean ± SEM, **P < 0.01, ***P < 0.005, ****P < 0.001). (*D*, *E*) Follicle counts in serial sections of PND3 Amhr2 knockout heterozygous (*D*) and homozygous (*E*) mice pup ovaries treated once a day for 4 d with gandotinib (40 mg/kg), SP600125 (45 mg/kg), CYC-116 (50 mg/kg), ruxolitinib (20 mg/kg), or vehicle control (*n* = 6 per group, mean ± SEM, **P < 0.01, ***P < 0.005, ****P < 0.001). PND7, postnatal day 7; PND12, postnatal day 12.

MIS is a potent suppressant of folliculogenesis that works by inhibiting both primordial follicle activation and the development of preantral follicles, resulting in the progressive loss of antral follicles and contraception (5, 8). As a proof of concept that the candidate MISR2 agonists we identified may be effective in inhibiting folliculogenesis in vivo, we evaluated their ability to reduce the abundance of developing follicles in neonatal rat pups, which have a large, coordinated wave of primordial follicle activation and growth after birth. The rat pups (PND2, *n* = 8 per group) were treated with daily intraperitoneal injections of MIS (3 mg/kg) or candidate agonists at doses previously established in the literature such as gandotinib (20 mg/kg) (21), SP600125 (30 mg/kg) (22), CYC-116 (50 mg/kg) (23), and ruxolitinib (20 mg/kg) (24) for 10 d (Fig. 6*A*). Rat ovaries were collected at day 10 and follicle counts were performed as previously described (Fig. 6*B*) (7, 25). Primordial follicle numbers were unaffected by treatments, while all four candidate agonists and MIS significantly reduced primary follicle numbers, and only MIS, gandotinib, CYC-116, and ruxolitinib significantly inhibited secondary and antral follicle counts, with SP600125 showing a trend of a modest decline (Fig. 6*C*). These data mirror the more pronounced activity of CYC-116 and gandotinib compared to SP600125 in both in vitro and ex vivo assays.

To confirm that the in vivo ovarian suppressive effects of drugs were specific to MISR2 agonism and not a result of toxicity to growing follicles, we treated neonatal transgenic mice bearing homozygous or heterozygous *Misr2* deletions with these candidates. Any effect of a drug in an *Misr2^−/−^* mouse would be attributed to either the mechanism of action of their intended target (e.g., JNK2, JAK2, Aurora kinase A/B) or nonspecific toxicity. We found that similar to the rat model, treatment of Misr2^+/−^ heterozygous neonatal mice (which have intact MISR2 signaling) with MIS, CYC-116, gandotinib, and ruxolitinib led to a statistically significant decrease in primary and secondary, whereas SP600125 did not (Fig. 6*D*). In contrast, neither the drugs nor MIS affected follicle counts in the Misr2^−/−^ homozygous knockout mouse littermates, confirming these drugs are dependent on MISR2 to elicit ovarian suppression, and suggesting the decrease in growing follicles is not the result of nonspecific toxicity to follicles (Fig. 6*E*).

## Discussion

There is an important need to expand the armamentarium of modulators of folliculogenesis that could be used for various aspects of women’s health. We have shown that recombinant MIS protein can be an effective contraceptive in a number of mammalian models; its unique mechanism of action (7) makes it ideally suited for several related applications such as the protection of ovarian reserve during exposure to chemotherapy (5) or the synchronization of follicles to enhance response to ovarian stimulation during assisted reproduction (8). However, biologics, such as the recombinant MIS protein, are less compatible with long-term use for contraception, given their high cost, their requirement to be administered by injection on a frequent schedule, and their relatively low stability. To address these issues, we aim to develop an orally active small molecule that can recapitulate the properties of MIS by pharmacologically agonizing its receptor, MISR2.

Herein we provide a proof of concept that we can screen for potent MISR2 agonists using a COS7 cell line transfected transiently with the MISR2 signaling machinery (*MISR2*/*ALK2*/*SMAD1*) and a BRE-luc luminescent reporter. We screened a repurposed drug library of 5,440 small molecules, which resulted in 16 hits, 9 of which were subsequently confirmed to significantly induce luciferase activity in an MISR2-dependent manner. Among these, four were selected (gandotinib, CYC-116, ruxolitinib, and SP600125) for further evaluation based on their ability to induce luciferase at pharmacologically relevant doses with a favorable biological activity and toxicity profile in the rat urogenital ridge Mullerian duct regression assay.

The identification of SP600125 by this screen provided compelling evidence of the validity of the method, given prior reports of its MIS-mimetic activity, which was independent from its intended inhibitory effect on JNK2 (10). Structurally, many of the initial hits in this screen could be assigned to 2 categories: flavonoids and pyrazole-/pyrrolopyrimidine-like. Many flavonoids were initially detected as hits, however, few produced dose-dependent activation in the secondary screen, suggesting that their potency may be too low to warrant further evaluation. In contrast, the four hits that we chose to further characterize, the pyrazole-/pyrrolopyrimidine-like compounds SP600125, gandotinib, CYC-116, and ruxolitinib, could significantly induce luciferase in the low micromolar range, and they displayed specific activity in the fetal rat urogenital ridge Mullerian duct regression assay. Interestingly, both gandotinib and ruxolitinib are structurally related JAK2 inhibitors, and both contain a core pyrrolopyrimidine with an adjacent alpha or beta pyrazole ring (26, 27). Similarly, CYC-116 and SP600125 also contain the pyrazole or analogous structure with a same-group substitution from the periodic table (22, 28). Further studies are needed to confirm the direct physical binding of these compounds to MISR2; we speculate that this binding site resides in the intracellular kinase domain of the receptor, which is truncated in the MISR2v3 splice variant.

While most candidate MISR2 agonists identified herein have not been studied in the context of folliculogenesis, ruxolitinib has recently been reported to cause activation and apoptosis of primordial follicles through inhibition of JAK1 (29). However, STAT3, the downstream target of JAK1, was not repressed by ruxolitinib (29). Furthermore, no significant differences were observed in the number of preantral follicles; rather, the distribution of primordial follicles was shifted away from follicles with squamous granulosa cells to those with a portion of cuboidal cells, a phenotype that we also reported in MIS-treated ovaries (7). We therefore speculate that the ovarian phenotype described with ruxolitinib treatment could be due in part to MISR2 agonism.

To further validate the ability of all four candidate compounds—SP600125, gandotinib, CYC-116, and ruxolitinib—to elicit MISR2 signaling in the ovary, we analyzed the expression of genes that we previously reported to be regulated by MIS in the granulosa cells of follicles: *Smad6*, *Igfbp5*, *Id2*, and *Id3* (7). The activity of the candidate compounds at 1 µM was generally equivalent to a high concentration of MIS (10 µg/mL) at inducing downstream genes in postnatal day 2 ex vivo mouse ovarian cultures. However, once translated in vivo, we found differential bioactivity of these compounds with SP600125 showing weaker activity compared to the other candidates.

While the commercial development of SP600125 as a JNK2 inhibitor and CYC-116 as an Aurora kinase-B inhibitor was abandoned, gandotinib and ruxolitinib, which are structurally related JAK2 inhibitors, are approved for human use. Gandotinib was evaluated in a phase II clinical trial (NCT01594723) for myeloproliferative neoplasms, myelofibrosis, polycythemia vera, and essential thrombocythemia, including patients who failed treatment with ruxolitinib; initial trial results suggest clinical benefits (30). Ruxolitinib, commercialized under the brand names of Jakafi and Jakavi, is approved for use in intermediate to severe myelofibrosis (31), polycythemia vera when the patient is resistant or intolerant to hydroxyurea (32), and graft-versus-host disease (33) and as a topical cream under the brand Opzelura for the treatment of mild to moderate atopic dermatitis (34). To our knowledge, reproductive toxicities have not been reported during the clinical toxicological evaluation of ruxolitinib and gandotinib. We speculate that the short toxicological evaluations may not capture ovarian suppression by MISR2 agonists, given our findings that MIS takes more than 30 d to visibly inhibit follicle maturation in adult female mice (5, 8). The evidence of ovarian suppression in vivo with gandotinib and ruxolitinib presented in the current study should warrant a careful examination of the potential side effects of these drugs on the fertility of women who use them chronically. Of particular concern is the potential exposure of female fetuses to these drugs if administered in pregnant patients, given our recent report that the activation of the MIS pathway during the development of the female reproductive tract leads to severe uterine hypoplasia and infertility (6).

The drugs identified herein warrant further investigation as tool compounds to design new therapeutic molecules with enhanced specificity to MISR2 and loss of activity of their original intended targets, such as JNK2 for SP600125 (22), JAK2 for gandotinib and ruxolitinib (21, 35), and Aurora kinase-A and -B for CYC-116 (23). Future studies examining long-term treatments of adults mice, including mating studies, will be needed to confirm in vivo contraceptive effects of these candidate MISR2 agonists. Furthermore, for MISR2 agonists to become viable contraceptives it will be key to confirm that they recapitulate key features of the recombinant MIS protein such as reversibility (5, 8), maintenance of healthy estrogen levels (5), long-term safety, absence of risk to pregnancy or fetus, and ensure that they do not have adverse effects on other organs expressing MISR2 such as the uterus (6) and the pituitary gland (36–38), given contraceptive treatments are usually taken for years, starting after puberty.

Screens and assays such as the ones described herein can be used to identify and prioritize modulators of MIS signaling or drugs aimed at modulating preantral follicle maturation via other targets. Notably, agonists of MISR2 hold promise not only in contraception but also in the protection of fertility during chemotherapy (5), the synchronization of follicles for assisted reproduction (8), and several other clinical applications (39) thanks to the unique suppressive mechanism of action of the MIS/MISR2 pathway in preantral follicles (7).

## Conclusion

Overall, these data suggest that SP600125, gandotinib, CYC-116, and ruxolitinib are agonists of MISR2 capable of suppressing the ovaries and thus represent tool compounds that could be formulated or modified to serve as modulators of folliculogenesis with a broad range of clinical application in women’s health.

## Materials and Methods

### Small-Molecule Screen and Dose–Response Luciferase Assays.

To identify small molecules that activate MISR2, we used a modified BMP-responsive element luciferase (BRE-luc) reporter assay as previously described (10). Briefly, activation of MISR2 signaling induces the BRE promoter, which drives the transcription of the firefly luciferase reporter.

On day 1, COS7 cells were plated in 10 cm culture dishes (6 × 10^6^ cells/dish) (Corning Life Sciences) and transfected with four plasmids incorporating essential components of the MIS signaling pathway (Genescript): MISR2 (V1 OHu22327 or V3 OHu56242), ALK2 (OHu16005D), ALK3 (OHu18274D), ALK6 (OHu12264D), SMAD1 (OHu20018D), SMAD5 (OHu23633D), SMAD9 (OHu08552D), and BRE-luc (11). In total, 25.5 mg plasmids (each plasmid 6.35 mg) were transfected with 153 µL Fugene6 (Promega) (mass/volume ratio of 1:6) in each dish. The transfection efficiency was confirmed to be greater than 80% by transfecting an identical plate with a Green fluorescent protein (GFP) expression plasmid (PCDNA3-eGFP-N1).

In the primary screen, the expression plasmids (MISR2v1, ALK2, and SMAD1 complementary DNA (cDNA) in a PCDNA3.1 backbone) and the BRE-luc reporter construct were transiently co-transfected into COS7 cells and plated at a density of 1.5 × 10^3^ cells per well in 384-well flat bottom plates and grown for 24 h. The screened repurposing library of 5,440 compounds originated from the Broad Institute’s Center for the Development of Therapeutics drug repurposing hub (https://clue.io/repurposing) (10), where the data generated herein was also deposited (40). Drugs were added in a volume of 100 nL at a 50 µM final concentration in DMSO into 30 µL cell medium and cultured for 24 h at 37 °C. The luciferase activity was measured using the Steady-Glo Luciferase Assay System (Promega) according to the manufacturer’s instructions. A hit was considered significant when the luminescence reading was 3 SDs above the plate’s mean. Compounds were tested in duplicate on separate plates and recombinant MIS (16) (10 µg/mL) was used as a positive control to confirm induction on each plate.

In the secondary screen, a dose–response was used to quantify activity in the presence of an active *MISR2v1* isoform or the inactive *MISR2v3* splice variant; we compared luciferase induction across a range of 10 doses titrated as 2-fold dilutions starting from 100 µM. For transfection, identical plates were generated using a transfection mix (*ALK2*, *SMAD1*, *BRE-Luc*) and containing either the cDNA of *MISR2v1* or *MISR2v3* as the fourth plasmid. Next, 24 h after transfection, COS7 cells were detached with Cellstriper (Corning) and plated into 384-well white plates (Thermo Fisher Scientific) (1.5 × 10^4^ cells/well) precoated with compounds. Furthermore, 24 h after plating, the luciferase signal was measured with Steady-Glo (Promega) and detected by the Envision luminometer (PerkinElmer).

### Determination of the Activity Score and Hits.

Each compound’s activity score was calculated using the following equation:

$$N\left(x\right)=CR+\frac{x-<cr>}{<sr>-<cr>}(SR-CR),$$

where *N* is the normalized activity value, *x* is the measured raw signal of a well, *<cr>* is the median of the measured signal values of the central reference (DMSO control), *<sr>* is the median of the measured signal values of the scale reference (inhibitor or agonist control), *CR* is the desired median normalized value for the central reference (0), and *SR* is the desired median normalized value for the scale reference (–100 or 100). Genedata was used to calculate these values. The activity threshold was set at (positive or negative) 3 times the SD of the DMSO control, the direction corresponding to activation or inhibition. Each compound was given one of three designations depending on the compound’s activity for each replicate. Compounds were classified as “active” if the mean of both replicates was equal to or more than the activity threshold. Compounds were classified as “inconclusive” if 1 of 2 replicates was equal to or less than the activity of the threshold but the mean of both replicates was above the activity threshold. Compounds were classified as “inactive” if neither of the replicates was equal to or less than the activity threshold. These classifications were done using Spotfire software.

### Cell Culture.

COS7 cells (African green monkey kidney cell line; American Type Culture Collection, Manassas, VA) were cultured in Dulbecco's Modified Eagle Medium (DMEM) supplemented with 10% female fetal bovine serum (to reduce bovine MIS), 2 mM L-glutamine, 100 U/mL penicillin, and 100 µg/mL streptomycin in a humidified 5% CO_2_ incubator at 37 °C.

### Plasmid Combination Optimization Assays.

COS7 cells were plated at a density of 10,000 cells per well in 96-well plates for secondary screens. Immediately after plating, the cells were transfected with *MISR2v1*, *ALK2/ALK3/ALK6*, *SMAD1/5/9*, and the BRE-luc reporter construct using the FuGene6 transfection reagent (Promega). Two days later, the cells were treated with MIS, SP600125 (Calbiochem), DMSO vehicle control (Thermo Fisher Scientific), or mock purified MIS vehicle control for 24 h. Luciferase activity was measured using the Steady-Glo Luciferase Assay System (Promega) according to the manufacturer’s instructions. Plates were read in a Wallac Victor2 luminometer (PerkinElmer).

### MIS Proteins.

The recombinant human MIS (rhMIS) protein was produced in our laboratory as previously described (16). Briefly, a CHO-K1 clone stably transfected with an *LR-MIS* expression vector was grown in HYPERflasks (Corning Life Sciences). The serum-free conditioned culture medium was collected after 72 h incubation and concentrated 10 to 20× using a tangential flow over a size exclusion membrane. The concentrated medium was incubated with Sepharose beads (Thermo Fisher Scientific) preconjugated with an anti-human MIS monoclonal antibody (clone 6E11). Bound rhMIS was eluted with a glycine-buffered solution (pH = 2.9). The eluates were dialyzed for 4 h against a 10× volume of phosphate buffered saline (PBS) and the rhMIS concentrations were adjusted to 1.2 mg/mL. All purified proteins were tested for bioactivity in the rat urogenital ridge bioassay and stored at −80 °C until use. As a vehicle control, a mock purification was performed, which recapitulated all purification steps, but used media conditioned by untransfected CHO-K1, and thus devoid of MIS.

### Animals.

This study was performed in accordance with the animal research protocol (2009N000033 and 2014N000275) approved by the Massachusetts General Hospital Institutional Animal Care and Use Committee. Animals were housed in standard conditions (12 h light/12 h dark) and were provided food and water ad libitum. Timed pregnant Sprague-Dawley rats (E14.5) (Envigo) were used to test the bioactivity of compounds in ex vitro cultures of fetal urogenital ridges as previously described (16, 17). Both the Sprague-Dawley rats and C57/B6 PND2 female mouse pups (Charles River Laboratories) were used for ex vivo ovarian culture bioassays, as previously described (7). Adult Friend leukemia virus B (FVB) female mice (Charles River Laboratories) were used for primordial follicle culture. The Sprague-Dawley female rat pups (PND2) were used to confirm the biological activity of candidate agonists in vivo. The *Misr2*^+/−^ and *Misr2*^−/−^ C57/B6 transgenic mice were a CRE knock-in that disrupts the MISR2 gene (41), Amhr2^tm3(cre)Bhr^ strain 014245-UNC, provided by the Mutant Mouse Resource and Research Centers U42OD010924, and were injected starting on postnatal day 3 (PND3).

#### Urogenital ridge bioassay.

The rat urogenital ridge bioassay was conducted as previously reported (16, 17). Briefly, the female embryonic ridges were dissected from timed pregnant Sprague-Dawley rats (E14.5) and incubated on 2% agarose (wt/vol) at the air/media interface on agarose gel suspended in a CMRL 1066 medium (Corning Life Sciences) supplemented with 10% female fetal bovine serum (Biologos), 1% L-glutamine (Thermo Fisher Scientific), 1% fungizone (Thermo Fisher Scientific), and 5 nM testosterone (Sigma Aldrich). The small-molecule compounds dissolved in DMSO were added into the culture medium for testing and compared to DMSO (0.02% final volume) vehicle control, and rhMIS (10 µg/mL) for positive control. Tissues were incubated for 72 h, fixed, embedded in paraffin, and then sectioned and stained with hematoxylin (DAKO) and eosin (Sigma Aldrich) (H&E). The regression score of the Mullerian duct was evaluated by two experienced blinded observers under a light microscope. The consensus scores were recorded, ranging from grade 0 (no regression) to grade 5 (complete regression). The integrity of the Wolffian duct and the appearance of the tissue were used as an indicator of the toxicity of compounds.

### Ovarian Culture Bioassay.

The ovaries were dissected from mouse or rat female pups (PND2) and incubated in 6-well trans-well plates (Corning) over a DMEM/F12 medium (Thermo Fisher Scientific) supplemented with 0.1% Albumaxc (Thermo Fisher Scientific), 0.1% female fetal bovine serum (Biologos), 25.7 µg/mL transferrin (Sigma Aldrich), 0.05 mg/L L-ascorbic acid (Sigma Aldrich), and 1% penicillin/streptomycin (Thermo Fisher Scientific). After 48 h incubation, ovaries were collected. For expression analysis, the RNA (from 2 ovaries) was extracted for qPCR of the MIS signaling pathway genes. For RNAish (RNA-scope), the ovaries were fixed with 10% neutral buffered formalin and embedded, sectioned, and kept at 4 °C until staining.

### Primordial Follicle In Vitro Culture.

The ovaries were dissected from female FVB mice (4–6 wk) and dissociated mechanically through a 70 µm cell strainer (Corning Life Sciences) into a 35 mm culture dish (Corning Life Sciences) containing PBS. Primordial follicles were manually pipetted from the dish under a microscope based on a diameter of less than 50 µm and a flattened granulosa cell morphology by an experienced embryologist. Individual primordial follicles were separated into 20 µL droplets of Minimum Essential Medium (MEM) (Thermo Fisher Scientific) supplemented with 10% female fetal bovine serum (Biologos) and 1% penicillin/streptomycin (Thermo Fisher Scientific), covered with mineral oil (Thermo Fisher Scientific) at 0.1 µM for 1 h at both the beginning and the end of the incubation.

#### qPCR.

Total RNA was extracted from the cultured rat or mouse ovaries after 48 h incubation with the compounds using the Qiagen RNA extraction kit (Qiagen). There were three biological samples in each treatment group and three technical replicates for each sample. For each biological sample, RNA was extracted from both combined ovaries for cDNA synthesis. The SuperScript III First-Strand Synthesis System (Invitrogen) was used for cDNA synthesis according to the manufacturer’s instructions using random hexamers. The gene target primers were designed to span the exon–exon junctions or include large introns (*SI Appendix*, Table S1) to avoid amplification of the contaminating genomic DNA. The SYBR Green Supermix (Bio-Rad) was used for PCR amplification on the Bio-Rad CTX96 real-time PCR detection system for quantification. Expression levels relative to Glyceraldehyde-3-phosphate dehydrogenase (*Gapdh*) were calculated using the cyclic threshold (Ct) values logarithmically transformed using the 2-^ΔCt^ function, and all the relative expression levels were normalized according to the average value of three biological samples in the control group.

### Histomorphology and Follicle Counts.

The dissected ovaries were fixed in 10% neutral buffered formalin (Sigma Aldrich) at room temperature overnight. The fixed ovaries were processed through serial ethanol, xylene, and paraffin baths in the automated tissue processor (Leica). The paraffin-embedded ovaries in blocks were cut serially into 6 µm sections, and every fifth section of the whole ovary was stained with H&E and analyzed for follicle counts. The follicles were categorized into primordial, primary, secondary, and antral follicles based on the follicle size as well as the shape and number of the surrounding layer(s) of granulosa cells (25). Only follicles with visible oocyte nuclei were counted. The total number of follicles counted for each category was then multiplied by a correction factor of 5 (the sampling rate) to be representative of the population of the entire ovary. The follicle counting was conducted independently by two experienced observers, and the average number was used for statistical analyses.

### RNAish.

The tissue section of the middle of the ovary was used for RNAish, performed as previously described (6). Briefly, paraffin-embedded ovaries were deparaffinized, dehydrated, and peroxidase-blocked and the epitope was retrieved by the target retrieval and protease plus reagents (ACD Bio) according to the manufacturer’s instructions. The ovarian sections were hybridized with predesigned or custom-designed probes (ACD Bio, *SI Appendix*, Table S2) spanning the mRNA of the target genes in the HybEZ hybridization oven (ACD Bio) for 2 h at 40 °C. The slides were then processed for standard signal amplification steps, and a red chromogen development was performed using the RNAscope 2.5HD (Red) detection kit (ACD Bio). The slides were then counterstained in 50% hematoxylin (Dako) for 2 min, air-dried, and cover-slipped with EcoMount (BioCare).

#### Immunofluorescence staining of ovarian sections.

The largest diameter of the ovary was used for immunofluorescence staining. Deparaffinization in xylene and rehydration in serial alcohols was conducted before tissue antigen retrieval was conducted by parboiling the ovarian section in 10 mM sodium citrate (pH 6.0) for 20 min. The tissues were blocked in 3% bovine serum albumin in tris buffered solution + 0.1% Tween (TBST) and incubated with primary antibody against DDX4 (1:1,000, Cell Signaling Technology) overnight at 4 °C. The tissue sections on the slide were then washed 3 times (10 min each) with TBST and incubated with horseradish peroxidase–conjugated secondary antibodies (1:5,000) (Alexa Fluor 555-conjugated donkey anti-rabbit IgG antibody, Thermo Fisher Scientific) at room temperature for 1 h and washed 3 times (10 min each) in TBST, then cover-slipped with a Vectashield mounting medium containing DAPI (Vector Laboratories).

### In Vivo Treatment.

#### Rats.

Rat pups (PND2) were injected intraperitoneally with rhMIS (3 mg/kg), gandotinib (20 mg/kg, Selleckchem), SP600125 (30 mg/kg, Selleckchem), CYC-116 (50 mg/kg, Selleckchem), or ruxolitinib (20 mg/kg, MedchemExpress) once per day for 10 d. At the end of treatment, necropsy was performed and the ovaries were dissected, fixed, processed, embedded, and H&E-stained for follicle count.

#### In vivo treatment of Misr2^−/−^ and Misr2^±/-^ transgenic mice.

Misr2/Amhr2Cre knock-in mice were purchased from the Mutant Mouse Regional Resource Centers (strain B6, 129S7- *Amhr2^tm3(cre)Bhr/Mmnc^*, back-crossed with C57BL/6J). Tail genotyping of the Misr2-cre knock-in and wild-type mice was done with the REDExtract-*N*-Amp Tissue PCR Kit (Sigma) with the previously described sets of primers (42). Misr2^cre/cre^ males had retained Mullerian ducts confirming the loss-of-function of Misr2. Misr2^cre/cre^ and Misr2^+/cre^ transgenic female mice (PND3) were injected intraperitoneally with rhMIS (3 mg/kg), gandotinib (20 mg/kg, Selleckchem), SP600125 (30 mg/kg, Selleckchem), CYC-116 (50 mg/kg, Selleckchem), or ruxolitinib (20 mg/kg once per day for 4 d, then killed by decapitation. The ovaries were processed, and follicle counts were performed as described above.

### Statistical Analyses.

A one-way ANOVA was used to compare the relative expression of target mRNA by qPCR. The zygote diameters in the fluorescent images were measured with Image J and analyzed using Fisher’s exact test to compare the percentages of activated follicles. The statistical analyses were conducted using Prism software (GraphPad version 8.0), with *P* < 0.05 regarded as statistically significant. A two-way ANOVA was conducted on luciferase dose–response experiments.

## Supplementary Material

Supplementary File

## Data Availability

All small molecules screen data are included in the following link: https://repurposing.broadinstitute.org/AssayResultsViewer, and in the *SI Appendix*.
