# Supplementary material for: A screen of repurposed drugs identifies AMHR2/MISR2 agonists as potential contraceptives
Source: Proc Natl Acad Sci U S A. 2022 Apr 5;119(15):e2122512119. doi: 10.1073/pnas.2122512119 (PMC9169708; doi:10.1073/pnas.2122512119)
Supplement: Supplementary File [file pnas.2122512119.sapp.pdf]

FigS1

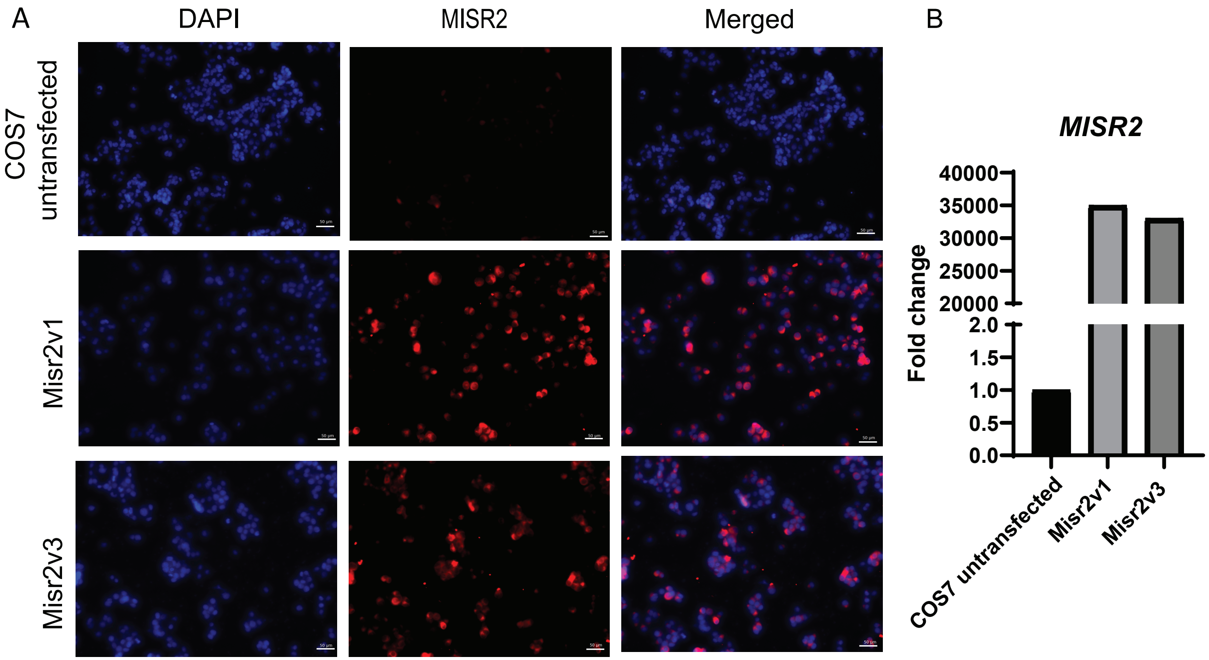

Figure S1: Validation of MISR2v3 protein and mRNA expression.
A) Immunofluorescence of MISR2 in COS7 untransfected or transfected with MISR2v1 or MISR2v3.
B) qPCR validation of MISR2 mRNA expression in MISR2v1 and MISR2v3 transfected COS7 cells.

FigS2

A

Atom Pairs -Tanimoto Structural Correlation

	Diosmetin	PPY-A	Gandotinib (LY2784544)	Apigenin	Linifanib (ABT-869)	Chrysin	Crizotinib	AG 494	CYC116	SP600125	Quercetin	INH1	ADX 10059 hydrochloride	Clobenpropit dihydrobromide	PJ 34 hydrochloride	Ruxoalitinib (INCB018424)
Diosmetin		0.293	0.138	0.642	0.233	0.558	0.158	0.293	0.233	0.253	0.627	0.266	0.174	0.069	0.266	0.073
PPY-A	0.282		0.241	0.268	0.257	0.271	0.219	0.251	0.236	0.272	0.223	0.381	0.175	0.107	0.313	0.121
Gandotinib (LY2784544)	0.146	0.196		0.117	0.213	0.110	0.251	0.111	0.290	0.109	0.119	0.190	0.133	0.167	0.180	0.187
Apigenin	0.909	0.297	0.152		0.248	0.741	0.143	0.338	0.144	0.294	0.619	0.272	0.167	0.083	0.257	0.083
Linifanib (ABT-869)	0.282	0.302	0.130	0.297		0.251	0.175	0.267	0.204	0.298	0.199	0.359	0.178	0.129	0.310	0.090
Chrysin	0.864	0.306	0.156	0.950	0.306		0.141	0.356	0.151	0.329	0.506	0.288	0.182	0.078	0.280	0.087
Crizotinib	0.182	0.208	0.146	0.163	0.184	0.167		0.122	0.232	0.113	0.135	0.185	0.162	0.173	0.137	0.178
AG 494	0.265	0.167	0.149	0.242	0.225	0.212	0.159		0.166	0.272	0.313	0.332	0.198	0.105	0.275	0.105
CYC116	0.143	0.149	0.136	0.150	0.174	0.154	0.143	0.237		0.179	0.130	0.255	0.172	0.187	0.217	0.187
SP600125	0.345	0.184	0.163	0.370	0.250	0.385	0.146	0.188	0.162		0.257	0.311	0.151	0.105	0.375	0.147
Quercetin	0.913	0.282	0.146	0.909	0.282	0.864	0.182	0.265	0.143	0.345		0.206	0.150	0.066	0.216	0.066
INH1	0.222	0.220	0.146	0.235	0.191	0.242	0.156	0.194	0.231	0.182	0.222		0.239	0.157	0.331	0.118
ADX 10059 hydrochloride	0.206	0.175	0.156	0.219	0.175	0.226	0.195	0.212	0.154	0.200	0.206	0.206		0.114	0.153	0.077
Clobenpropit dihydrobromide	0.200	0.171	0.178	0.212	0.171	0.219	0.163	0.206	0.150	0.194	0.200	0.235	0.219		0.117	0.157
PJ 34 hydrochloride	0.250	0.186	0.192	0.265	0.214	0.273	0.128	0.294	0.225	0.482	0.250	0.154	0.167	0.162		0.136
Ruxoalitinib (INCB018424)	0.184	0.244	0.143	0.194	0.214	0.200	0.295	0.128	0.225	0.177	0.184	0.184	0.167	0.132	0.180	

Maximum Common Substructure - Tanimoto Structural Correlation

B

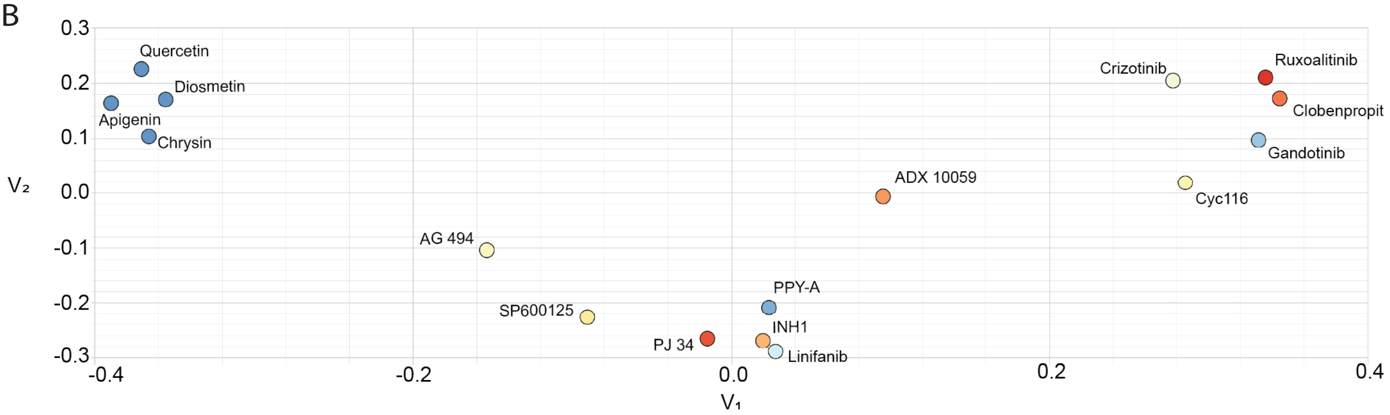

Figure S2: Structural homology of selected candidate agonists.
A) Atom pair and maximum common substructure Tanimoto correlation.
B) Principal component analysis clustering using Tanimoto correlation values

FigS3

A.

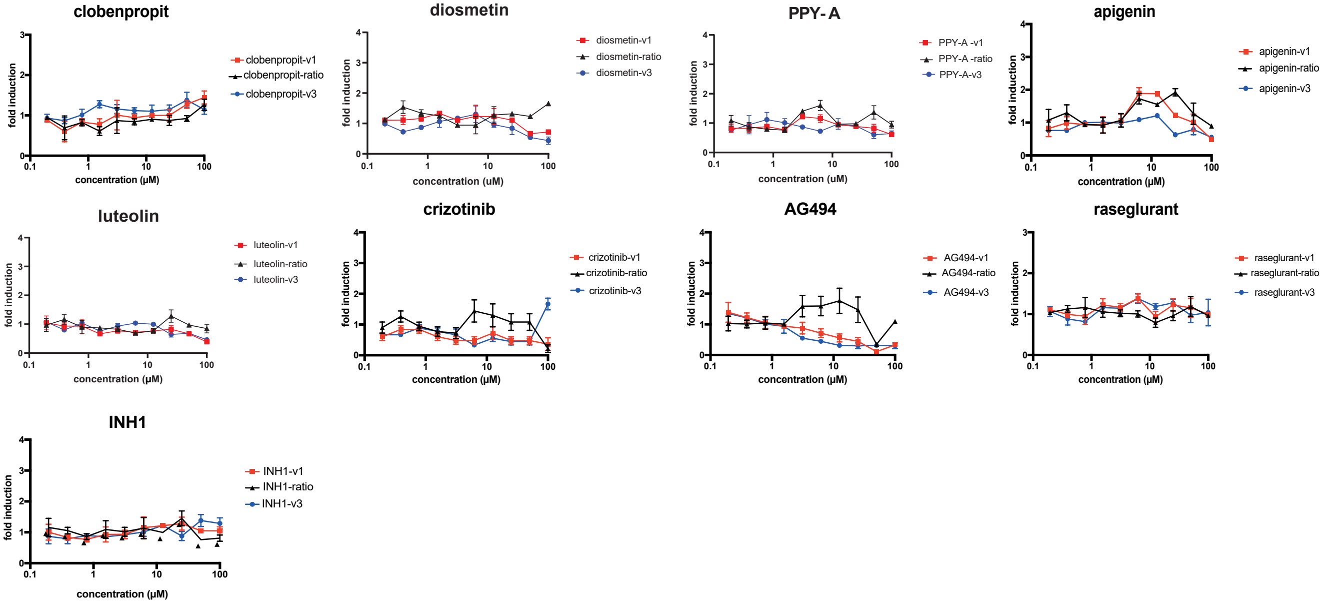

B.

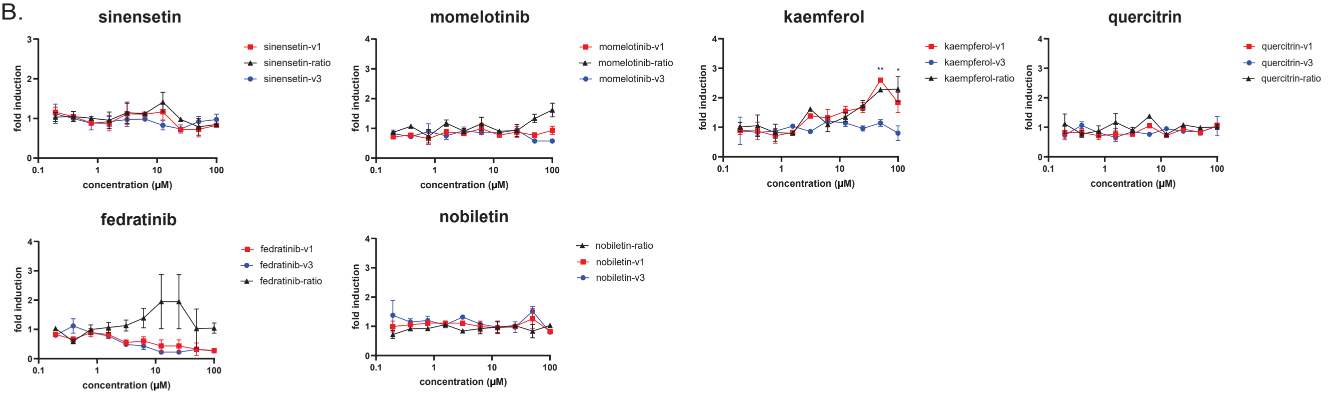

C.

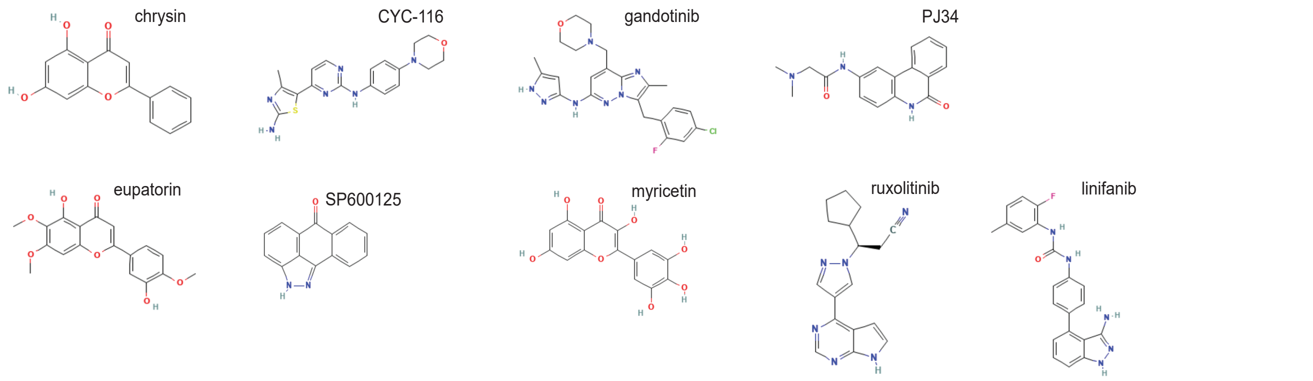

Figure S3 : Activity and structure of screen hits and related chemical compounds
A-B) Luciferase induction compared to vehicle control is plotted across a 10-point range (195nM to 100µM) of compounds tested in the first screen (A), compounds added based on their chemical proximity to the initial library compounds (B), in COS7 cells transfected with either MISR2v1 in red or MISR2v3 in blue (in addition to ALK2/SMAD1/Bre-luc) and incubated for 24h. The MISR2v1/MISR2v3 signal ratio is also plotted in black (replicated twice, mean ± SEM). C) Structure of selected candidate agonists.

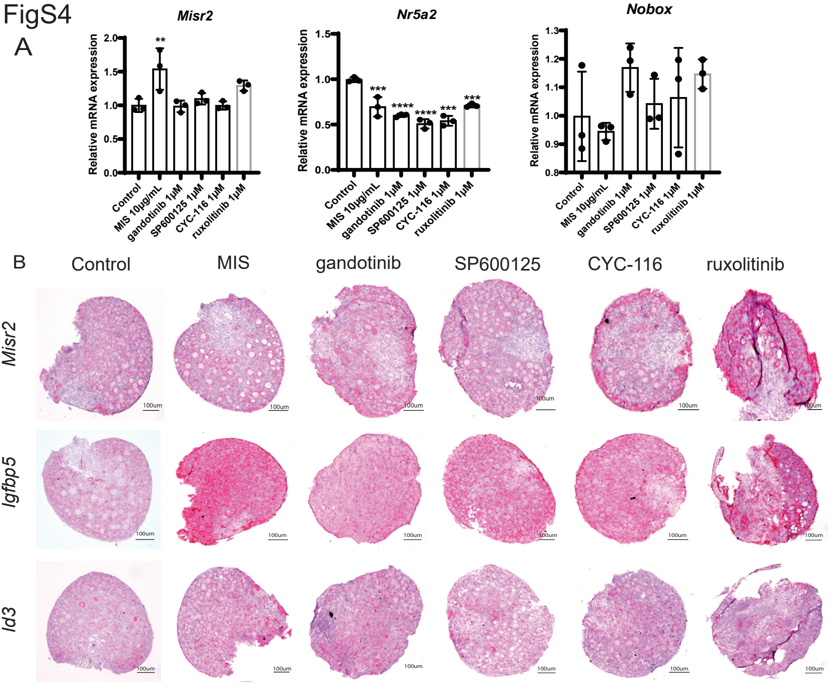

Figure S4: MISR2 agonists regulate MIS downstream targets but not MISR2 nor oocytes specific genes. PND2 mice ovaries were incubated for 48h with 1µM of candidate MISR2 agonists gandotinib, SP600125, CYC-116, ruxolitinib or MIS at 10µg/ml as a positive control and vehicle control (DMSO). A) qPCR evaluation of Misr2, the granulosa cell marker Nr5a2 and the oocyte marker Nobox (n=3 per group, mean ± SD; **P < 0.01, ***P<0.005, ****P<0.001). B) Pattern of expression of *Misr2*, *Id3*, and *Igfbp5* evaluated by RNAish (representative ovary section, n=1 per treatment)

FigS5

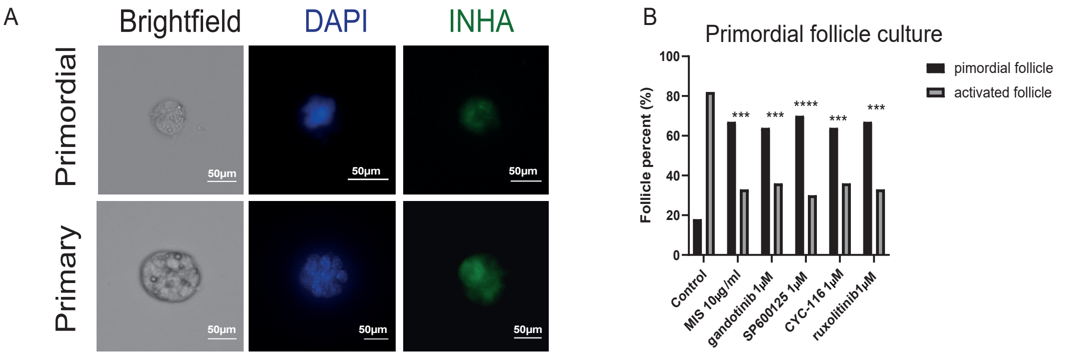

Figure S5: Effect of MISR2 agonists on primordial follicles activation
A-B) Follicle activation was scored based on cytomorphology, confirmed by INHA immunofluorescence (N=33 per group) (A) in primordial follicles from adult mice cultured in vitro for 48h with 1μM of candidate MISR2 agonists gandotinib, SP600125, CYC-116, ruxolitinib or MIS at 10μg/ml as a positive control and DMSO as a vehicle control (B).

Table S1: Probes used for RNA in situ hybridization (RNAscope)

Gene name	Species	Accession number	Detection region	ACD catalog number
Igfbp5	Mouse	NM_010518.2	746 - 1999	Cat No. 425731
Id3	Mouse	NM_008321.2	8 - 848	Cat No. 445881
Misr2	Mouse	NM_144547.2	914 - 1809	Cat No. 489821

Table S2: Primers designed for qPCR

Gene name	Forward	Reverse	NCBI	Species
Id2	TCTGGGGGATGCTGGGCACC	GCTTGGGCATCTCCCGGAGC	NM_010496.3	Mouse
ID2	CACAAGGAATTGCCCAATC	GAACTGGTATTTATTTGGG	NM_013060.3	Rat
Id3	CAGGGTCCCAAGCGAACGG	TTGCCACTGACCCGGTCGTC	NM_008321.2	Mouse
ID3	TCAGCTTAGCCAGGTGGAAA	TGAGCTCAGCTGTCTGGATCG	NM_013058.2	Rat
Igfbp5	GAGACAGGAATCCGAACAAG	GAGGGCTTACACTGCTTTC	NM_010518.2	Mouse
IGFBP5	CCAAGCACACTCGCATTT	GGTCAGATTCCTGTCTCATCT	NM_012817	Rat
Smad6	CTGTCTCTTCTCCGAACGGG	CTTGAGCAGCGAGTACGTGA	NM_008542.3	Mouse
SMAD6	AAGCCACTGGATCTGTCCGA	GCCCTGAGGTAGGTCGTAGA	NM_001109002.2	Rat
Misr2	GCGGCAGCACAAGTATC	CATCCTTGCATCTCCACTTG	NM_001356575.1	Mouse
Nobox	TAGGAAAATGCGGGGCCAG	GCTGTGTGCACTCTACAGGT	NM_130869.3	Mouse
Nr5a2	TCATGCTGCCCAAAGTGGAGA	TGGTTTTGGACAGTTCGCTT	NM_001159769.2	Mouse
